# Stomata Traits Diversity in Wild Accessions of *Coffea racemosa* and *C. zanguebariae* from Mozambique

**DOI:** 10.3390/plants14223466

**Published:** 2025-11-13

**Authors:** Niquisse José Alberto, Larícia Olária Emerick Silva, Gianluca Luongo, Armando Francisco Saide, Tércio Felisberto Horácio, Sitina José José, Salito Alexandre Bernardo, José C. Ramalho, Fábio Luiz Partelli

**Affiliations:** 1 Department of Agrarian and Biological Sciences (DCAB), Federal University of Espírito Santo/UFES, Rod. BR 101 Norte, Km 60, Bairro Litorâneo, São Mateus 29932-540, Espírito Santo, Brazil; niquissealberto@gmail.com; 2 Higher Polytechnic Institute of Mecuburi, Campus Tottoto, Mecubúri 3100, Nampula, Mozambique; 3 United Nations Industrial Development Organization-UNIDO (IET/AGR/RAP), Wagramer Str. 5, Wien 1220, Australia; g.luongo@unido.org; 4 Faculty of Health Sciences, Lúrio University, Bairro Marrere 3100, Nampula , Mozambique; arsaide@unilurio.ac.mz; 5 Faculty of Natural Sciences, Lúrio University, Bairro Eduardo Mondlane, Pemba 3200, Cabo Delgado, Mozambique; tercio.horacio@unilurio.ac.mz (T.F.H.); sjose1@unilurio.ac.mz (S.J.J.); 6 Instituto Oikos, Ilha de Ibo 3205, Cabo Delgado, Mozambique; agronomo.ibo@istituto-oikos.org; 7 Forest Research Center (CEF), Associate Laboratory TERRA, School of Agriculture (ISA), University of Lisbon (ULisboa), Quinta do Marquês, Av. República, 2784-505 Oeiras and Tapada da Ajuda, 1349-017 Lisboa, Portugal; 8 GeoBioSciences, GeoTechnologies and GeoEngineering Unit (GeoBioTec), NOVA School of Science and Technology (FCT/UNL), Monte de Caparica, 2829-516 Caparica, Portugal

**Keywords:** *Coffea racemosa*, *Coffea zanguebariae*, leaf anatomy, stomatal density and morphology

## Abstract

Estimated climate change scenarios demand robust coffee cultivars tolerant to supra-optimal temperatures, water deficit, diseases, and other stresses. Wild *Coffea* species represent important genetic resources for resilience. The study of variations in morphological structures associated with transpiration control, such as stomata, represents an important approach for identifying genotypes with greater stress tolerance. This study evaluated stomatal density and morphology in 48 wild accessions, 24 of *Coffea racemosa* and 24 of *C. zanguebariae*, from provinces of Mozambique. Leaf samples provided microscopic images to assess stomatal traits: density (SD), polar diameter (PD), equatorial diameter (ED), stomatal functionality (SF), and leaf dry mass. Principal components were analyzed for all 48 accessions and separately by species. Mean distribution independence was tested with the Mann–Whitney test (*p* < 0.05). Results revealed inter- and intraspecific variation. The ability to distinguish accessions varies with the set of traits and species. A significant negative correlation between ED and SF was shared by both species, suggesting a conserved functional pattern. This study discusses the differences in stomatal traits between wild and commercial coffee species and aspects related to possible alterations of stomatal structures during their adaptation to climate change. Additionally, it points to accessions with potential use in genetic breeding programs to increase stomatal function and the possible adaptation of new cultivars.

## 1. Introduction

The genus *Coffea* belongs to the Rubiaceae family and comprises at least 130 species [1]. Wild *Coffea* species show a morphological, genetic, and biochemical diversity, with some of which displaying better resistance to pests, diseases, and environmental constraints than *C. arabica* L. (Arabica coffee, with a wide number of cultivars) and *C. canephora* Pierre ex Froehner (Robusta coffee, dominated by Conilon and Robusta cultivars) that support the global coffee market [2,3].

Climate changes and global warming poses considerable challenges to coffee crop sustainability. The greater speed of temperature rise, climate instability, and change in rainfall temporal and spatial distribution [4,5] can endanger the adaptability of new cultivars in recent decades [6,7,8]. Under these scenarios, wild species constitute important genetic resources to develop new cultivars with greater resilience to climate change. In fact, underutilized wild coffee species, such as *C. liberica* Hiern., *C. eugenioides* Moore, *C. racemosa* Lour., and *C. zanguebariae* Lour., may have potential for economic exploration, helping to diversify the genetic base of coffee production, while offering new possibilities in genetic breeding programs [1,9]. The use of wild species may represent a key strategy to promote leaf anatomical modifications that improve plant adaptation to stress conditions, such as a reduction in stomatal density and an increase in stomatal efficiency [10].

In Mozambique, *C. arabica* has recently begun to be cultivated in regions with high edaphoclimatic diversity [11], but native species *C. zanguebariae* and *C. racemosa* (known as Inhambane coffee) [4,12] have been used for a long time. Despite the cultivation of native species, there remains the need for genotypes with higher productivity and adaptation to microregions and genetic diversity studies on the potential use of this material in breeding programs to guide strategies to develop new cultivars [13].

Commercial transactions in the German market in south-east Africa traded *C. zanguebariae* in 1893. Its cultivation began on Ibo Island (northeastern Mozambique) in 1920 as an indigenous variety of southern Tanzania, northern Zimbabwe, and northern Mozambique as a substitute for *C. arabica* [14,15]. The cultivation of *C. zanguebariae* on Ibo Island occurs in regions with an average annual rainfall of approximately 950 mm and an average annual temperature of 26 °C [4]. On the other hand, *C. racemosa* was described by Loureiro [12] based on a specimen of a herbarium sample from Mozambique. The originally identified species has been lost, being replaced by accessions from the district of Massingir (Gaza province) in southwestern Mozambique [15]. The name Inhambane coffee also commonly refers to *C. racemosa*, whose aroma quality has long been recognized [16], being related to the region of accession collection for this species: southern Mozambique [4]. This species cultivation occurs in regions with an average annual rainfall of approximately 807 mm and an average annual temperature of 22.9 °C [4].

Studies on the genetic divergence of coffee accessions, almost exclusively regarding *C. arabica* and *C. canephora*, are usually focused on agronomic phenotypic traits and ecophysiological characteristics, especially leaf anatomical variations [17,18,19]. This type of trait enables to assess the phenotypic plasticity or the adaptability of genotypes under intense environmental modifications [20]. These studies are essential to guiding plant breeding toward anatomical modifications that may favor the potential adaptation capability of new cultivars.

Variations in stomatal density and morphology have been reported in accessions of *C. canephora* and *C. arabica* [18,21,22], likely associated with distinct adaptation ability, even within species, although these species are more domesticated than *C. zanguebariae* and *C. racemosa*. In fact, stomatal control plays an important role in regulating gas exchange and water loss in plants, being directly influenced by variations in stomatal density and morphology [23]. Under environmental stress conditions such as drought, high temperatures, or excessive light, the ability to adjust stomatal density and aperture can lead to significant improvements in plant water-use efficiency [24]. Thus, genotypes with greater efficiency in this control tend to exhibit better physiological performance and increased resilience, especially in environments with irregular water availability or higher solar radiation intensity. Therefore, the analysis of stomatal traits represents a valuable tool for selecting more adaptable genetic materials, contributing significantly to breeding strategies in the face of climate change scenarios.

Thus, this study will provide newly information regarding stomatal traits of *C. racemosa* and *C. zanguebariae* accessions from Mozambique, and establish the following hypotheses: (i) stomatal density and morphology differ between *C. racemosa* and *C. zanguebariae* individuals, and (ii) differences in stomatal density and morphology occur at both interspecific and intraspecific levels.

## 2. Results

The evaluation of leaf traits indicated differences in the mean values among *C. racemosa* accessions (Table 1), as reflected by the wide amplitude of values among accessions, which exceeded the minimum significant difference. The equatorial diameter of the stomata (ED) had the highest coefficient of variation (CV) (60.5%), indicating greater variation in the equatorial diameter of stomatal cells among leaves of the same accessions. In contrast, stomatal density (SD) showed the lowest CV (25.2%), indicating the lowest variation in the structures between leaves of the same plant.

Regarding dry mass (DM), the Cr2 genotype (0.15 g) showed a higher average than others, indicating a greater accumulation of biomass (Table 1). Conversely, Cr8, Cr10, Cr12, Cr15, Cr19, Cr23, and Cr24 (0.03 g) showed lower DM values, possibly associated with less favorable growth conditions or less efficient genetics.

In general, Cr8 showed the highest SD value (395.86), while Cr9 had the lowest mean value (116.25). This difference showed the possibility of adding Cr9, Cr17, and Cr20 into a group with statistically lower SD means than the others (Table 1). Regarding ED, the accessions Cr2, Cr1, Cr22, Cr21, Cr25, Cr17, Cr14, Cr15, Cr23, Cr24, and Cr9 showed higher absolute means, significantly differing from the other accessions. Moreover, ED showed the highest CV, evincing high variation, even in leaves of the same plant. Regarding polar diameter (PD), Cr 21, Cr2, Cr22, Cr15, and Cr17 showed a higher mean diameter than the others.

Considering stomatal functionality (SF), Cr1 and Cr2 had lower absolute means (Table 1), statistically differing from Cr8 and Cr10, which presented higher values.

As regards leaf traits in *C. zanguebariae* accessions, CV values (Table 2) remained relatively below those for *C. racemosa* (Table 1). Additionally, the amplitude between SD, ED, and SF means remained below the minimum significant difference, showing no statistically significant differences among the means.

The highest DM was observed in Cz14 (0.23), a value that statistically differed from those for Cz11, Cz16, Cz22, Cz19, and Cz18, which had the lowest means of leaf dry mass (Table 2). Regarding PD, Cz3 (31.84) showed the highest value, indicating greater stomatal cell length, with a mean statistically similar to the other 13 accessions, which showed a mean above 27.88.

In general, the general means among the species showed that *C. zanguebariae* has a dry mass and stomatal size practically twice as large as *C. racemosa*. However, *C. racemosa* has higher density and stomatal functionality.

**Figure 1:**
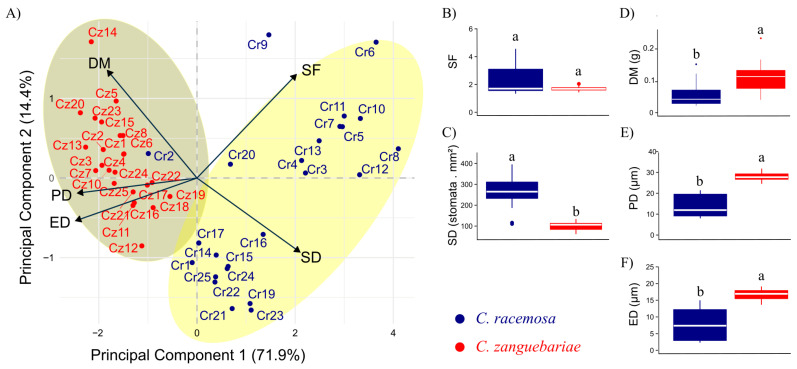
Analysis of principal components (**A**) and boxplots (**B**–**F**) regarding five leaf traits in 48 accessions of *Coffea* belonging to two species: *C. racemosa* (Cr) and *C. zanguebariae* (Cz) sampled in Mozambique. DM: dry mass of leaves (g); SD: stomatal density (number of stomata mm^2^); PD: polar diameter of the stomata (μm); ED: equatorial diameter of the stomata (μm); SF: stomatal functionality. Different letters between the boxes (boxplot) indicate an independent distribution in the species according to the Mann–Whitney test at a significant level of 5%.

Considering both species together, the decrease in the initial variables for two principal components retained 86% (PC1 = 71.9% and PC2 = 14.4%) of the variability in the original dataset (Figure 1A). PD and ED showed a higher correlation with PC1, whereas DM, SF, and SD showed more balanced relations with the two components (despite a greater variation in the first component).

In the dispersion of accessions based on the evaluated traits, SD and SF constitute the main criteria for separating *C. racemosa* and *C. zanguebariae* accessions, as by projected them on opposite sides of the biplot. Accessions Cr8, Cr6, Cr10, and Cr12 stand out among those with the highest SD and SF. A single *C. racemosa* accession, Cr2, showed characteristics that brought it closer to *C. zanguebariae* accessions in the grouping, notably due to its lower SF and SD than the other accessions of its species.

This study obtained distinct subgroups of *C. racemosa* (Figure 1A): one with a higher SF and lower SD (Cr6 and Cr9), a second with higher SF and SD (Cr11, Cr10, Cr7, Cr5, Cr13, Cr4, Cr3, Cr8, and Cr12), a third with low SF and SD (Cr20 and Cr2), and finally, a subgroup with higher SD and lower SF (Cr17, Cr16, Cr14, Cr15, Cr1, Cr25, Cr24, Cr22, Cr19, Cr21, and Cr23).

The *C. zanguebariae* accessions showed a more clustered pattern, without clear subgroups (Figure 1A). Still, Cz 14 was separated from the others with the highest DM and lowest SD.

The SF failed to distinguish the two species (Figure 1B), but the SD displayed an independent distribution across species according to the Mann–Whitney test (Figure 1C). Except for Cr2 and Cr20, no other *C. racemosa* accession showed SD values as low as those of *C. zanguebariae.* DM behaved otherwise, as, despite the independent distribution, values overlapped between at least 50% of the accessions (Figure 1D). PD (Figure 1E) and ED (Figure 1F) showed a pattern similar of that of DM as regards lower values in *C. racemosa*, but with no or little overlap.

**Figure 2:**
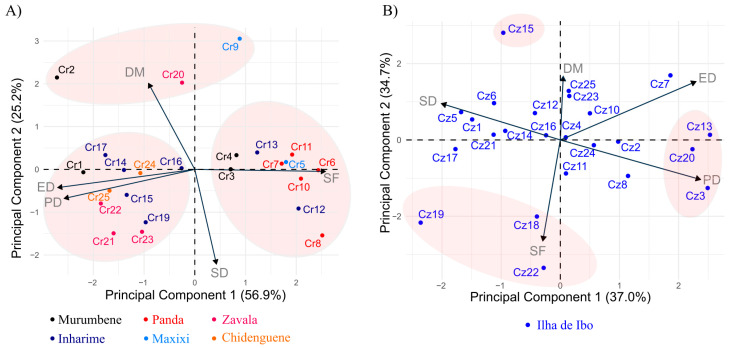
Principal component analysis for five leaf traits in 48 accessions of *Coffea* belonging to two species: (**A**) *C. racemosa* (Cr) and (**B**) *C. zanguebariae* (Cz), sampled in Mozambique. DM: dry mass of leaves (g); SD: stomatal density (number of stomata mm^2^); PD: polar diameter of the stomata (μm); ED: equatorial diameter of the stomata (μm); SF: stomatal functionality (PD/ED).

The individual dispersion of accessions in relation to the principal components by species (Figure 2) showed that the first two components represented 82.1% of the total variation for *C. racemosa* (Figure 2A) and 71.7% of the total variation for *C. zanguebariae* (Figure 2B). For *C. racemosa*, accessions such as Cr6, Cr11, Cr8, Cr12, Cr5, Cr7, and Cr13 showed higher SF, while others, such as Cr2, Cr20, and Cr9, showed similar DM. However, Cr9 had a higher SF and lower SD than the other two accessions. Conversely, accessions Cr1, Cr17, Cr14, Cr24, Cr16, Cr15, Cr25, Cr21, Cr22, Cr23, and Cr19 showed a higher PD and ED and lower SF. Notably, these groups of accessions did not correspond to the collection of single districts, since it included accessions from different districts (Figure 2A). The principal components showed a different pattern as regards *C. zanguebariae*: the first component gathered PD, ED, and SD, whereas the second component was strongly related to SF and DM (Figure 2B). However, as observed for *C. racemosa*, a lower discrimination occurred for DM and ED than for the other traits, with a total variation of less than two standard deviation units from the mean. Accessions Cz13, Cz20, and Cz23 had higher ED and DP and lower SD. Accessions Cz22, Cz19, and Cz18 showed higher SF, in which the SD of Cz19 exceeded that of the other two. Cz15 showed a higher DM and a high SD. Variations in SD, ED, and PD dispersed the other *C. zanguebariae* accessions.

In *C. racemosa*, significant correlations were observed among stomatal traits (Table 3), notably a strong positive correlation between the ED and PD (r = 0.97; *p* < 0.01), as well as negative correlations between the ED and SF (r = −0.87; *p* < 0.01) and between the PD and SF (r = −0.77; *p* < 0.01). In *C. zanguebariae*, SD was negatively correlated with ED (r = −0.48; *p* < 0.05), and the ED also showed a negative correlation with SF (r = −0.61; *p* < 0.05). The negative correlation between ED and SF was the only significant relationship shared by both species, suggesting a conserved functional pattern.

## 3. Discussion

This study evaluated traits related to the density and size of stomata in 48 accessions of *Coffea* of two wild species. The results showed greater variations among *C. racemosa* accessions than among *C. zanguebariae* ones (Figure 1A). However, this research found groups of accessions with different aptitudes for a certain number of traits within each species. Such studies of traits related to stomata can provide insights associated with the potential for adaptation of a given accession or species. Several studies of other species reported this phenomenon, obtaining evidence of possibly exploiting genetic effects linked to the adaptation of stomatal structures to increase the resilience of plants to climate change [25,26,27]. In fact, stomatal size and density are key determinants of maximum stomatal conductance, g*s* [28,29]. For example, in a wide number of species it was observed that elevated air CO_2_ concentration can alter stomatal traits and function, namely by reducing stomatal opening and reducing SD, thus reducing g*s* [30,31,32]. These findings evidence the potential and importance of prospecting stomatal traits from the wild accessions evaluated in the present study, aiming to incorporate climate change resilience into future coffee cultivars.

Regarding the potential of stomatal traits to discriminate accessions, the four traits (PD, ED, SD, and SF) were important for distinguishing species (vector length in Figure 1A). However, within species, SF contributed the most to discriminating accessions. This may occur because of the more pronounced negative correlation between SF and PD or ED in *C. racemosa* compared with *C. zanguebariae*, in addition to the strong positive correlation between PD and ED in *C. racemosa* compared with the weaker correlation observed in *C. zanguebariae* (Table 3). In other words, in *C. racemosa*, higher PD values result in higher ED values and consequently lower SF values, whereas in *C. zanguebariae*, this tendency is less evident. The ratio between PD and ED, which determines SF, can vary under water deficit conditions, as demonstrated by Melo et al. [33]. In an assessment of the Siriema coffee cultivar under both water-stressed and non-stressed conditions, notable contrasts in ED and PD were observed: PD increased while ED decreased under water deficit, resulting in a rise in SF from 1.46 to 1.75 [33]. These findings support the use of SF as a parameter to infer the potential adaptation of genotypes to water-limited environments.

In *C. racemosa*, several accessions stood out for their combination of high stomatal density (SD) and elevated stomatal functionality (SF), traits that may be associated with greater gas exchange capacity and faster stomatal responses. Studies about SF in model plants reported that SF (as result of stomatal size structures) may represent an important indicator of plant responses to environmental stresses, as higher SF values suggest an enhanced capacity for CO_2_ uptake per stomatal opening [34]. This characteristic can contribute to increased rates of CO_2_ assimilation while minimizing water loss during stomatal activity. Accessions such as Cr8, Cr6, and Cr10 exhibited a combination of high SD and SF, simultaneously, suggesting greater efficiency in transpiration regulation under variable environmental conditions. Studies in rice, wheat, and *Arabidopsis* have shown that smaller, more elongated stomata (with higher PD/ED ratios) promote better control over transpiration and faster closure [35,36].

Accordingly, accessions Cr6 and Cr8, which combine small stomata with high functionality, may reflect a similar anatomical mechanism with adaptive potential under drought. In contrast, *C. zanguebariae* accessions showed larger stomata, lower density, and reduced functionality. Despite the higher dry mass observed in some accessions (such as Cz14), this set of traits may indicate a lower capacity for transpiration control, associated with a greater number of stomata per leaf and a higher potential for water loss during stomatal opening [37].

Findings in *Coffea* spp. also reported some changes promoted by elevated air CO_2_, but with species-dependent impacts on SD reduction, and an almost invariant g*s* [21,38]. Furthermore, stomatal traits can also respond to other environmental conditions in *Coffea* spp. Studies on *C. arabica* have showed that SD tends to increase with the increase in photosynthetically active radiation [39]. Also, high temperatures can promote changes in a species-dependent manner. These included increases in SD and stomatal index in *C. canephora* cv. Conilon CL153, whereas the opposite patterns were observed in *C. arabica* genotypes [17,40]. These findings were suggested to reflect different temperature sensing of stomata among *Coffea* species/genotypes, with implications for the control of transpiration (together with stomata opening extend) and, thus, of water-use efficiency.

High levels of anatomical differences among species of *Coffea*, including *C. racemosa*, *C. arabica*, and *C. liberica,* also point to relevant phenotypic plasticity [41]. Studies on variation in stomatal structures between accessions within species have also reported variability in *C. arabica* and *C. canephora* [18,22,42]. However, studies on the intraspecific variation in traits related to the anatomy of stomata involving *C. racemosa* or *C. zanguebariae* remain scarce in the literature. Regarding the possibility of relating phenotypic variation to phenotypic plasticity (the ability of an organism to modify its phenotype in response to environmental changes), Valladares et al. [43], in a review on methodologies for quantifying phenotypic plasticity, emphasized the nonlinearity in the relationship between variation and plasticity. Although phenotypic variation is a prerequisite for phenotypic plasticity within a population, conclusions about the potential plasticity of the accessions investigated here can only be drawn after studies assess their performance across multiple environments.

In a study with 43 genotypes of *C. canephora* in Brazil, Dubberstein et al. [22] showed the mean values of 282 stomata per mm^2^ of leaf area, with a mean equatorial diameter of 25.7 μm and a mean polar diameter of 16.9 μm. Pérez-Molina et al. [18], in a study with genotypes of *C. arabica* var. Catucaí, indicated an average stomatal density of 158 stomata per mm^2^ of leaf area. Considering these values as a reference, the stomatal density values in this study showed that *C. zanguebariae* had a lower stomatal density than the *C. arabica* and *C. canephora* commercial cultivars, and a stomatal functionality close to that of *C. canephora* (Table 2). *C. racemosa* has a stomatal density close to *C. canephora* (262.8 stomata per mm^2^) and greater stomatal functionality (2.27) (Table 1). This may indicate greater adaptive plasticity and water-use efficiency in *C. racemosa*, reinforcing its potential as a donor of alleles for drought tolerance in breeding programs. Indeed, this species has already been identified as a source of desirable traits, such as early fruit maturation, drought resistance [44,45], and greater tolerance to pests [46].

The correlation analyses support these interpretations. In both species, the negative correlation between stomatal equatorial diameter (ED) and stomatal functionality (SF) indicates a conserved functional pattern, in which narrower stomata tend to be more elongated, favoring greater control over transpiration. This relationship aligns with patterns reported in species adapted to water-stressed environments [29,47]. Similar structural adaptations were also observed in *Pancratium maritimum*, a coastal species exhibiting unique inter-stomatal connections composed of pectins and extensins, which appear to modulate stomatal aperture and enhance water-use control under harsh conditions [48]. Therefore, the levels of modification observed in leaf structures, such as stomatal density and morphology, may represent promising targets for future research aiming to deepen the understanding of the transmission potential of these traits in breeding crosses and to evaluate the enhancement of adaptive capacity in these genotypes.

Genetic divergence studies between different species of *Coffea* have shown a considerable difference between *C. racemosa* × *C. zanguebariae* [4] and between *C. racemosa* × *C. arabica* [3]. However, comparative studies between *C. zanguebariae* and commercial species seem scarce. The differences among *C. racemosa* accessions in this study corroborate the possibility of high intraspecific genetic diversity, including stomatal density and morphology traits (Figure 1A). On the other hand, the smallest intraspecific variation among *C. zanguebariae* accessions may suggest a lower genetic variation for these traits. However, it should be underlined that this study had limitations regarding the scope of its collection area, especially for *C. zanguebariae*. Thus, complementary studies that integrate anatomical, molecular, physiological, and environmental data will be essential for a more accurate assessment of the genetic variability of *C. racemosa* and *C. zanguebariae*. This integrated approach may support more effective strategies for the development of cultivars with enhanced resilience to climate-related stresses.

## 4. Materials and Methods

### 4.1. Plant Material and Characterization of the Experimental Area

From 2023 to 2024, randomly distributed samples of wild populations were collected at their natural sites in southern Mozambique for *C. racemosa* in Chidenguele (Gaza province), Murumbene, Maxixi, Inharrime, Panda, and Zavala (all from Inhambane province), and in northeastern Mozambique for *C. zanguebariae* on Ibo Island (Cabo Delgado province), with the specific coordinates identified at the collection sites gives in Table 4. Specimens from the National Herbarium were also searched at the Institute of Agrarian Research of Mozambique (created in 1967) to obtain additional information on habitat, vegetation, environment, morphology, and cultivation. The sampled plants are commonly collected by local harvesters and used either for self-consumption or for sale in local markets.

### 4.2. Dry Mass and Leaf Anatomy

Samples of *C. racemosa* were collected in December 2023 and those *C. zanguebariae* in February 2024, all during the fruit maturation phase. All leaf samples were collected from different positions around the plant, using the third and/or fourth pair of leaves (counted from the apex) from plagiotropic branches in the middle-third part of the plant canopy. For leaf dry mass estimation (DM, g), eight leaves were used per plant (each plant corresponds to an accession). For the determination of dry mass, the leaves were kept in a forced-air oven at 60 °C until a constant mass was observed. The final value of DM was obtained from the mean of eight sampled leaves.

For anatomical analysis, two additional leaves from each accession were used. To obtain epidermal impressions, a solution of colorless enamel was fixed in the central part of the leaf for five minutes. After drying, they were mounted on semi-permanent slides with transparent adhesive tape. These epidermal leaf imprints were then examined and documented, using an optical microscope (Optika Microscopes Italy (Ponteranica, Italy), Model: B-290TB, magnification = 40×) and photographed with an attached camera. Five distinct and randomly selected points on the abaxial surface of the leaf epidermis were sampled for each slide, totaling 10 images per plant. Thus, the data of this study considered 480 images of leaf epidermis (240 for each wild species). The images were evaluated using Anati Quanti2 software (version 2.0) [49] for the following traits: stomatal density (the number of stomata per mm^2^—SD); polar diameter of the stomata (PD, μm); equatorial diameter of the stomata (ED, μm); and stomatal functionality (SF). They were expressed using the ratio between polar and equatorial diameters, following Castro et al. [50].

### 4.3. Statistical Analysis

Data on dry mass (DM) and stomatal parameters were expressed as means and standard deviations for each accession (24 of *C. racemosa* and 24 *C. zanguebariae*). The minimum significant distance to define the differences between means was estimated according to HSD Tukey’s test at a 5% significance level (α = 0.05). Based on the means, the five variables were standardized for principal component analysis (PCA). The first two principal components were used to construct boxplot charts. A PCA was first carried out for all 48 accessions. Then, subsequent PCAs were performed for each accession. The independence between the species datasets was tested using the Mann–Whitney test at a 5% significance level.

Pearson’s correlation coefficients between pairs of traits within each species were also estimated and tested using the *t*-test at a 5% significance level. The analyses and construction of graphs were carried out on R Core Team [51] based on the functions available in the stats, extra fact, and ggplot2 packages.

## 5. Conclusions

The wild species *C. racemosa* and *C. zanguebariae* differ in their stomatal density and morphology. Individuals of *C. racemosa* tend to have higher density and stomatal functionality compared to those of *C. zanguebariae*. At the intraspecific level, *C. racemosa* accessions show more pronounced variation than *C. zanguebariae* accessions. The findings confirm the two initial hypotheses of this research and show wild accessions with potential use in coffee breeding programs.

## Figures and Tables

**Table 1:** Evaluation of leaf dry mass (DM, g), stomatal density (SD, number of stomata mm^2^), equatorial diameter (ED, μm), polar diameter (PD, μm), and stomatal functionality (SF = PD/ED) from leaf samples collected in 24 accessions of *Coffea racemosa*. Each value represents the mean ± standard deviation (*n* = 10).

Genotype	DM (g)	SD (n. stom.mm^2^)	ED (μm)	PD (μm)	SF (=PD/ED)
Cr1	0.09 ± 0.02	288.65 ± 43.09	14.45 ± 1.07	19.76 ± 2.15	1.37 ± 0.08
Cr2	0.15 ± 0.04	219.92 ± 20.73	14.73 ± 0.67	20.24 ± 1.58	1.37 ± 0.08
Cr3	0.08 ± 0.02	329.89 ± 37.38	4.74 ± 0.68	11.18 ± 0.82	2.38 ± 0.18
Cr4	0.06 ± 0.02	246.31 ± 43.74	4.64 ± 0.54	11.67 ± 0.72	2.55 ± 0.41
Cr5	0.04 ± 0.02	261.16 ± 37.98	2.93 ± 0.33	8.32 ± 1.06	2.86 ± 0.45
Cr6	0.04 ± 0.02	233.67 ± 37.06	2.57 ± 0.55	11.27 ± 0.63	4.56 ± 0.99
Cr7	0.05 ± 0.01	252.91 ± 51.05	3.02 ± 0.26	9.59 ± 0.54	3.20 ± 0.33
Cr8	0.03 ± 0.01	395.86 ± 53.87	2.29 ± 0.23	8.54 ± 0.66	3.76 ± 0.55
Cr9	0.12 ± 0.05	116.25 ± 37.48	3.37 ± 0.64	8.34 ± 0.93	2.53 ± 0.46
Cr10	0.03 ± 0.01	285.90 ± 42.38	2.60 ± 0.24	9.12 ± 0.48	3.52 ± 0.27
Cr11	0.05 ± 0.02	266.35 ± 27.91	2.52 ± 0.31	7.98 ± 1.02	3.19 ± 0.35
Cr12	0.03 ± 0.01	320.11 ± 22.30	2.71 ± 0.45	8.10 ± 0.70	3.06 ± 0.61
Cr13	0.08 ± 0.03	290.30 ± 66.22	3.55 ± 0.67	9.21 ± 1.19	2.67 ± 0.55
Cr14	0.04 ± 0.01	195.73 ± 36.58	11.91 ± 0.99	19.36 ± 1.23	1.63 ± 0.10
Cr15	0.03 ± 0.01	237.52 ± 27.03	11.69 ± 0.76	20.62 ± 1.50	1.76 ± 0.06
Cr16	0.08 ± 0.02	305.45 ± 23.18	7.73 ± 0.87	12.19 ± 1.60	1.58 ± 0.10
Cr17	0.05 ± 0.02	186.93 ± 37.74	12.94 ± 1.03	20.23 ± 1.30	1.57 ± 0.07
Cr19	0.03 ± 0.01	303.49 ± 50.58	11.25 ± 0.69	17.62 ± 1.08	1.57 ± 0.08
Cr20	0.07 ± 0.02	119.39 ± 17.03	7.00 ± 0.67	11.33 ± 1.39	1.61 ± 0.08
Cr21	0.04 ± 0.01	327.44 ± 20.41	13.37 ± 1.26	21.65 ± 1.22	1.63 ± 0.09
Cr22	0.07 ± 0.01	312.29 ± 38.51	13.78 ± 0.94	20.72 ± 0.78	1.51 ± 0.11
Cr23	0.03 ± 0.01	321.09 ± 27.82	11.54 ± 0.8	18.29 ± 1.19	1.59 ± 0.09
Cr24	0.03 ± 0.01	202.33 ± 22.71	11.28 ± 0.66	17.19 ± 1.00	1.53 ± 0.10
Cr25	0.06 ± 0.02	288.1 ± 35.08	13.27 ± 0.34	19.13 ± 0.66	1.44 ± 0.05
Mean	0.058	262.79	7.91	14.23	2.27
Amplitude	0.12	276.47	12.44	13.67	3.19
CV (%)	54.74	25.24	60.54	36.37	40.01
DMS (5%)	0.10	208.92	6.98	2.06	1.87

CV—coefficient of variation; DMS—minimum significant distance (*n* = 8) according to Tukey’s test at a significance level of 5% (α = 0.05).

**Table 2:** Evaluation of leaf dry mass (DM, g), stomatal density (SD, number of stomata mm^2^), equatorial diameter (ED, μm), polar diameter (PD, μm), and stomatal functionality (SF = PD/ED) from leaf samples collected in 24 accessions of *Coffea zanguebariae*. Each value represents the mean ± standard deviation (*n* = 10).

Genotype	DM (g)	SD (n. stom.mm^2^)	ED (μm)	PD (μm)	SF (=PD/ED)
Cz1	0.14 ± 0.02	129.21 ± 27.41	15.94 ± 1.05	26.68 ± 2.45	1.67 ± 0.11
Cz2	0.13 ± 0.02	98.97 ± 18.69	17.73 ± 1.87	30.10 ± 2.75	1.71 ± 0.20
Cz3	0.10 ± 0.04	61.58 ± 13.91	17.99 ± 1.64	31.84 ± 1.95	1.78 ± 0.13
Cz4	0.11 ± 0.06	82.47 ± 10.18	16.56 ± 0.63	27.12 ± 2.63	1.64 ± 0.13
Cz5	0.16 ± 0.04	119.74 ± 33.19	15.53 ± 1.03	25.73 ± 1.07	1.66 ± 0.09
Cz6	0.13 ± 0.03	94.57 ± 25.50	16.06 ± 1.62	24.51 ± 1.44	1.54 ± 0.18
Cz7	0.12 ± 0.02	73.31 ± 24.59	19.48 ± 1.60	27.94 ± 2.63	1.44 ± 0.13
Cz8	0.11 ± 0.03	81.69 ± 16.62	17.05 ± 1.63	30.27 ± 1.59	1.79 ± 0.15
Cz10	0.12 ± 0.02	114.36 ± 13.91	18.04 ± 1.20	29.20 ± 1.97	1.63 ± 0.16
Cz11	0.08 ± 0.02	112.41 ± 17.19	16.79 ± 1.03	29.72 ± 2.32	1.77 ± 0.16
Cz12	0.05 ± 0.02	134.15 ± 19.26	17.83 ± 1.68	28.00 ± 1.71	1.58 ± 0.17
Cz13	0.13 ± 0.04	74.77 ± 11.36	19.03 ± 1.16	31.41 ± 1.50	1.66 ± 0.14
Cz14	0.23 ± 0.02	114.36 ± 24.97	15.40 ± 0.89	28.27 ± 3.43	1.85 ± 0.28
Cz15	0.17 ± 0.04	112.16 ± 19.26	17.20 ± 1.62	24.72 ± 2.63	1.45 ± 0.17
Cz16	0.07 ± 0.03	94.57 ± 18.11	16.78 ± 2.21	26.70 ± 1.85	1.61 ± 0.21
Cz17	0.08 ± 0.03	109.96 ± 17.96	15.09 ± 1.35	25.28 ± 2.40	1.68 ± 0.18
Cz18	0.05 ± 0.01	76.97 ± 11.59	15.12 ± 1.56	27.41 ± 2.06	1.83 ± 0.24
Cz19	0.06 ± 0.01	114.36 ± 13.91	13.63 ± 1.14	26.16 ± 2.22	1.93 ± 0.17
Cz20	0.15 ± 0.04	76.97 ± 11.59	18.33 ± 1.46	31.69 ± 2.18	1.74 ± 0.17
Cz21	0.09 ± 0.03	131.95 ± 21.99	16.39 ± 1.49	27.60 ± 2.37	1.69 ± 0.19
Cz22	0.06 ± 0.02	81.37 ± 14.84	14.52 ± 1.51	29.42 ± 2.28	2.05 ± 0.26
Cz23	0.16 ± 0.03	105.56 ± 22.71	17.53 ± 1.29	27.86 ± 1.65	1.60 ± 0.16
Cz24	0.11 ± 0.05	107.76 ± 16.23	17.55 ± 2.15	29.72 ± 2.15	1.72 ± 0.25
Cz25	0.12 ± 0.02	112.16 ± 16.23	18.06 ± 1.37	27.58 ± 1.94	1.53 ± 0.10
Mean	0.11	100.64	16.81	28.12	1.69
Amplitude	0.17	60.84	5.85	7.33	0.61
CV (%)	38.28	20.31	8.62	7.50	8.37
DMS (5%)	0.15	75.48	6.95	3.96	0.75

CV—coefficient of variation; DMS—minimum significant distance (*n* = 8) according to Tukey’s test at a significance level of 5% (α = 0.05).

**Table 3:** Matrices with Pearson’s correlation coefficients between five leaf traits in two species of *Coffea: C. racemosa* and *C. zanguebariae* in Mozambique. DM: dry mass of leaves (g); SD: stomatal density (stomata mm^2^); PD: polar diameter of the stomata (μm); ED: equatorial diameter of the stomata (μm); SF: stomatal functionality (μm).

*C. racemosa*					*C. zanguebariae*			
	SD	ED	PD	SF	SD	ED	PD	SF
DM	−0.34	0.16	0.03	−0.28	0.08	0.23	0.00	−0.27
SD		−0.06	−0.02	0.17		−0.28	−0.48 *	−0.16
ED			0.97 **	−0.87 **			0.51	−0.61 *
PD				−0.77 **				0.36

*, ** indicate correlation coefficients significantly different from zero according to the *t*-test at the significance levels of 5% and 1%, respectively.

**Table 4:** Localization of the collected of 24 accessions of *C. racemosa* (Cr) and 24 accessions of *C. zanguebariae* (Cz) from several regions in Mozambique.

Identification	Location	Location		Coordinate	Coordinate	Altitude
	Province	District	Species	S	E	m
Cr1	Inhambane	Murumbene	*C. racemosa*	23°31′56.01504″	35°23′37.41576″	61
Cr2	Inhambane	Murumbene	*C. racemosa*	23°31′56.01504″	35°23′37.41576″	61
Cr3	Inhambane	Murumbene	*C. racemosa*	23°31′56.01504″	35°23′37.41576″	61
Cr4	Inhambane	Murumbene	*C. racemosa*	23°31′55.18048″	35°20′39.58152″	61
Cr5	Inhambane	Maxixi	*C. racemosa*	23°49′55.2148″	35°20′55.21128″	18
Cr6	Inhambane	Panda	*C. racemosa*	24°3′27.6714″	34°44′17.44764″	156
Cr7	Inhambane	Panda	*C. racemosa*	24°3′27.6714″	34°44′17.44764″	156
Cr8	Inhambane	Panda	*C. racemosa*	24°3′27.6714″	34°44′17.44764″	156
Cr9	Inhambane	Maxixi	*C. racemosa*	23°49′55.2148″	35°20′55.21128″	18
Cr10	Inhambane	Panda	*C. racemosa*	24°3′27.6714″	34°44′17.44764″	156
Cr11	Inhambane	Panda	*C. racemosa*	24°3′27.6714″	34°44′17.44764″	156
Cr12	Inhambane	Inharime	*C. racemosa*	24°28′30.55764″	35°1′18.50124″	48
Cr13	Inhambane	Inharime	*C. racemosa*	24°28′30.55764″	35°1′18.50124″	48
Cr14	Inhambane	Inharime	*C. racemosa*	24°28′30.55764″	35°1′18.50124″	48
Cr15	Inhambane	Inharime	*C. racemosa*	24°28′30.55764″	35°1′18.50124″	48
Cr16	Inhambane	Inharime	*C. racemosa*	24°28′36.43428″	35°1′17.44608″	48
Cr17	Inhambane	Inharime	*C. racemosa*	24°28′36.43428″	35°1′17.44608″	48
Cr19	Inhambane	Inharime	*C. racemosa*	24°28′36.43428″	35°1′17.44608″	48
Cr20	Inhambane	Zavala	*C. racemosa*	24°30′23.7672″	34°59′57.51276″	30
Cr21	Inhambane	Zavala	*C. racemosa*	24°30′23.7672″	34°59′57.51276″	30
Cr22	Inhambane	Zavala	*C. racemosa*	24°30′23.7672″	34°59′57.51276″	30
Cr23	Inhambane	Zavala	*C. racemosa*	24°30′23.7672″	34°59′57.51276″	30
Cr24	Gaza	Chidenguele	*C. racemosa*	24°54′30.00276″	34°10′34.30524″	62
Cr25	Gaza	Chidenguele	*C. racemosa*	24°54′30.00276″	34°10′34.30524″	62
Cz01	Cabo Delgado	Ilha de Ibo	*C. zanguebariae*	12°20′38.11596″	40°35′26.55312″	14
Cz02	Cabo Delgado	Ilha de Ibo	*C. zanguebariae*	12°20′38.11596″	40°35′26.55312″	14
Cz03	Cabo Delgado	Ilha de Ibo	*C. zanguebariae*	12°20′38.11596″	40°35′26.55312″	14
Cz04	Cabo Delgado	Ilha de Ibo	*C. zanguebariae*	12°20′38.11596″	40°35′26.55312″	14
Cz05	Cabo Delgado	Ilha de Ibo	*C. zanguebariae*	12°20′38.11596″	40°35′26.55312″	14
Cz06	Cabo Delgado	Ilha de Ibo	*C. zanguebariae*	12°20′38.11596″	40°35′26.55312″	14
Cz07	Cabo Delgado	Ilha de Ibo	*C. zanguebariae*	12°20′38.11596″	40°35′26.55312″	14
Cz08	Cabo Delgado	Ilha de Ibo	*C. zanguebariae*	12°20′21.10908″	40°35′27.25908″	9
Cz10	Cabo Delgado	Ilha de Ibo	*C. zanguebariae*	12°20′21.10908″	40°35′27.25908″	9
Cz11	Cabo Delgado	Ilha de Ibo	*C. zanguebariae*	12°20′21.10908″	40°35′27.25908″	9
Cz12	Cabo Delgado	Ilha de Ibo	*C. zanguebariae*	12°20′52.28268″	40°35′36.91068″	14
Cz13	Cabo Delgado	Ilha de Ibo	*C. zanguebariae*	12°20′52.28268″	40°35′36.91068″	14
Cz14	Cabo Delgado	Ilha de Ibo	*C. zanguebariae*	12°20′52.28268″	40°35′36.91068″	14
Cz15	Cabo Delgado	Ilha de Ibo	*C. zanguebariae*	12°20′52.28268″	40°35′36.91068″	14
Cz16	Cabo Delgado	Ilha de Ibo	*C. zanguebariae*	12°20′52.28268″	40°35′36.91068″	14
Cz17	Cabo Delgado	Ilha de Ibo	*C. zanguebariae*	12°20′52.28268″	40°35′36.91068″	14
Cz18	Cabo Delgado	Ilha de Ibo	*C. zanguebariae*	12°20′15.62712″	40°35′4.00164″	12
Cz19	Cabo Delgado	Ilha de Ibo	*C. zanguebariae*	12°20′15.62712″	40°35′4.00164″	12
Cz20	Cabo Delgado	Ilha de Ibo	*C. zanguebariae*	12°20′15.62712″	40°35′4.00164″	12
Cz21	Cabo Delgado	Ilha de Ibo	*C. zanguebariae*	12°20′27.59388″	40°35′9.29292″	12
Cz22	Cabo Delgado	Ilha de Ibo	*C. zanguebariae*	12°20′28.8006″	40°35′8.18556″	12
Cz23	Cabo Delgado	Ilha de Ibo	*C. zanguebariae*	12°20′15.7938″	40°35′23.59536″	11
Cz24	Cabo Delgado	Ilha de Ibo	*C. zanguebariae*	12°20′15.7938″	40°35′23.59536″	11
Cz25	Cabo Delgado	Ilha de Ibo	*C. zanguebariae*	12°20′15.7938″	40°35′23.59536″	11

## Data Availability

The data is contained within the article.

## References

[1] Davis A.P., Rakotonasolo F. (2021). Six new species of coffee (*Coffea*) from northern Madagascar. Kew Bull..

[2] (2025). ICO-International Coffee Organization-Monthly Coffee Market Report. https://www.ico.org/documents/cy2024-25/cmr-0525-e.pdf.

[3] Tapaça I.D.P.E., Mavuque L., Corti R., Pedrazzani S., Maquia I.S., Tongai C., Partelli F.L., Ramalho J.C., Marques I., Ribeiro-Barros A.I. (2023). Genomic evaluation of *Coffea arabica* and its wild relative *Coffea racemosa* in Mozambique: Settling resilience keys for the coffee crop in the context of climate change. Plants.

[4] Davis A.P., Gargilo R., Almeida I.N.M., Caravela M.I., Denison C., Moat J. (2021). Hoot Coffee: The identity, climate profiles, agronomy, and beverage characteristics of *Coffea racemosa* and *C. zanguebariae*. Front. Sustain. Food Syst..

[5] Hassan W., Nayak M.A., Azam M.F. (2024). Intensifying spatially compound heatwaves: Global implications to crop production and human population. Sci. Total Environ..

[6] Chapman S.C., Chakraborty S., Dreccer M.F., Howden S.M. (2012). Plant adaptation to climate change: Opportunities and priorities in breeding. Crop Pasture Sci..

[7] Kromdijk J., Long S.P. (2016). One crop breeding cycle from starvation? How engineering crop photosynthesis for rising CO_2_ and temperature could be one important route to alleviation. Proc. R. Soc. B Biol. Sci..

[8] Palit P., Kudapa H., Zougmore R., Kholova J., Whitbread A., Sharma M., Varshney R.K. (2020). An integrated research framework combining genomics, systems biology, physiology, modelling and breeding for legume improvement in response to elevated CO_2_ under climate change scenario. Curr. Plant Biol..

[9] Davis A.P., Chadburn H., Moat J., O’Sullivan R., Hargreaves S., Nic L.E. (2019). High extinction risk for wild coffee species and implications for coffee sector sustainability. Sci. Adv..

[10] Pitaloka M.K., Caine R.S., Hepworth C., Harrison E.L., Sloan J., Chutteang C., Phunthong C., Nongngok R., Toojinda T., Ruengphayak R. (2022). Induced genetic variations in stomatal density and size of rice strongly affects water use efficiency and responses to drought stresses. Front. Plant Sci..

[11] Magalhães T.M., Reckziegel R.B., Paulino J. (2025). Soil organic carbon in tropical shade coffee agroforestry following land-use changes in Mozambique. Agrosys Geosci. Environ..

[12] Loureiro J. (1790). Flora Cochinchinensis.

[13] Navarini L., Scaglione D., Del Terra L., Scalabrin S., Mavuque L., Turello L., Nguenha R., Luongo G. (2024). Mozambican *Coffea* accessions from Ibo and Quirimba Islands: Identification and geographical distribution. AoB Plants.

[14] Bridson D.M., Polhill R.M., Verdcourt B. (1988). “Coffea” in Flora of Tropical East Africa, Rubiaceae.

[15] Bridson D.M., Pope G.V. (2003). “Coffe” in Flora Zambesiaca.

[16] Vasconcellos E. (1906). Catálogo da Exposição Colonial—Algodão, Borracha, Cacau e Café.

[17] Rodrigues W.P., Silva J.R., Ferreira L.S., Machado Filho J.A., Figueiredo F.A., Ferraz T.M., Bernado W.P., Bezerra L.B.S., Abreu D.P., Cespom L. (2018). Stomatal and photochemical limitations of photosynthesis in coffee (*Coffea* spp.) plants subjected to elevated temperatures. Crop Pasture Sci..

[18] Pérez-Molina J.P., de Toledo Picoli E.A., Oliveira L.A., Silva B.T., de Souza G.A., Santos Rufino J.L., Pereira A.A., Ribeiro M.F., Malvicini G.L., Turello L. (2021). Treasured exceptions: Association of morphoanatomical leaf traits with cup quality of *Coffea arabica* L. cv. “Catuaí”. Food Res. Inter..

[19] Silva L.O.E., Schmidt R., Almeida R.N., Feitoza R.B.B., Cunha M., Partelli F.L. (2023). Morpho-agronomic and leaf anatomical traits in *Coffea canephora* genotypes. Ciênc. Rural.

[20] Pompelli M., Martins S., Celin E., Ventrella M., DaMatta F. (2010). What is the influence of ordinary epidermal cells and stomata on the leaf plasticity of coffee plants grown under full-sun and shady conditions?. Braz. J. Biol..

[21] Ramalho J.C., Rodrigues A.P., Semedo J.N., Pais I.P., Martins L.D., Simões-Costa M.C., Leitão A.E., Fortunato A.S., Batista-Santos P., Palos I.M. (2013). Sustained photosynthetic performance of *Coffea* spp. under long-term enhanced [CO_2_]. PLoS ONE.

[22] Dubberstein D., Oliveira M.G., Aoyama E.M., Guilhen J.H., Ferreira A., Marques I., Ramalho J.C., Partelli F.L. (2021). Diversity of leaf stomatal traits among *Coffea canephora* Pierre ex A. Froehner genotypes. Agronomy.

[23] Haworth M., Marino G., Materassi A., Raschi A., Scutt C.P., Centritto M. (2023). The functional significance of the stomatal size to density relationship: Interaction with atmospheric [CO_2_] and role in plant physiological behaviour. Sci. Total Environ..

[24] Liao Q., Ding R., Du T., Kang S., Tong L., Li S. (2022). Stomatal conductance drives variations of yield and water use of maize under water and nitrogen stress. Agric. Water Manag..

[25] Chua L.C., Lau O.S. (2024). Stomatal development in the changing climate. Development.

[26] Lang P.L., Erberich J.M., Lopez L., Weiß C.L., Amador G., Fung H.F., Latorre S.M., Lasky J.R., Burbano H.A., Expósito-Alonso M. (2024). Century-long timelines of herbarium genomes predict plant stomatal response to climate change. Nat. Ecol. Evol..

[27] Xiao Z., Ma G., Bai X., Li J., Zhao M., Su L., Zhou H. (2024). The influence of leaf anatomical traits on photosynthesis in Catimor type Arabica coffee. Beverage Plant Res..

[28] Woodward F.I., Kelly C.K. (1995). The influence of CO_2_ concentration on stomatal density. New Phytol..

[29] Franks P.J., Beerling D.J. (2009). Maximum leaf conductance driven by CO_2_ effects on stomatal size and density over geologic time. Proc. Natl. Acad. Sci. USA.

[30] Woodward F.I. (1987). Stomatal index are sensitive to increase in CO_2_ from pre-industrial levels. Nature.

[31] Ainsworth E.A., Rogers A. (2007). The response of photosynthesis and stomatal conductance to rising [CO_2_]: Mechanisms and environmental interactions. Plant Cell Environ..

[32] Possell M., Hewitt C.N. (2009). Gas exchange and photosynthetic performance of the tropical tree *Acacia nigrescens* when grown in different CO_2_ concentrations. Planta.

[33] Melo E.F., Fernandes-Brum C.N., Pereira F.J., Castro E.M.D., Chalfun-Júnior A. (2014). Anatomic and physiological modifications in seedlings of *Coffea arabica* cultivar Siriema under drought conditions. Ciênc. Agrotec..

[34] Bertolino L.T., Caine R.S., Gray J.E. (2019). Impact of stomatal density and morphology on water-use efficiency in a changing world. Front. Plant Sci..

[35] Dittberner H., Korte A., Mettler-Altmann T., Weber A.P., Monroe G., Meaux J. (2018). Natural variation in stomata size contributes to the local adaptation of water-use efficiency in *Arabidopsis thaliana*. Mol. Ecol..

[36] Xiong Z., Dun Z., Wang Y., Yang D., Xiong D., Cui K., Peng S., Huang J. (2021). Effect of stomatal morphology on leaf photosynthetic induction under fluctuating light in rice. Front. Plant Sci..

[37] Ennajeh M., Vadel A.M., Cochard H., Khemira H. (2010). Comparative impacts of water stress on the leaf anatomy of a drought-resistant and a drought-sensitive olive cultivar. J. Hortic. Sci. Biotechnol..

[38] Rodrigues W.P., Martins M.Q., Fortunato A.S., Rodrigues A.P., Semedo J.N., Simões-Costa M.C., Pais I.P., Leitão A.E., Colwell F., Goulao L. (2016). Long-term elevated air [CO_2_] strengthens photosynthetic functioning and mitigates the impact of supra-optimal temperatures in tropical *Coffea arabica* and *C. canephora* species. Glob. Change Biol..

[39] Matos F.S., Wolfgramm R., Gonçalves F.V., Cavatte P.C., Ventrella M.C., DaMatta F.M. (2009). Phenotypic plasticity in response to light in the coffee tree. Environ. Exp. Bot..

[40] Rodrigues W.P., Vieira H.D., Teodoro P.E., Partelli F.L., Barbosa D.H.S.G. (2016). Assessment of genetic divergence among coffee genotypes by Ward-MLM procedure in association with mixed models. Genet. Mol. Res..

[41] Mauri R., Cardoso A.A., Silva M.M., Oliveira L.A., Avila R.T., Martins S.C., DaMatta F.M. (2020). Leaf hydraulic properties are decoupled from leaf area across coffee species. Trees.

[42] Alberto N.J., Ferreira A., Barros A.I.R., Aoyama E.M., Silva L.O.E., Rakocevic M., Ramalho J.C., Partelli F.L. (2024). Plant morphological and leaf anatomical traits in *Coffea arabica* L. cultivars cropped in Gorongosa Mountain, Mozambique. Horticulturae.

[43] Valladares F., Sanchez-Gomez D., Zavala M.A. (2006). Quantitative estimation of phenotypic plasticity: Bridging the gap between the evolutionary concept and its ecological applications. J. Ecol..

[44] Fazuoli L.C., Maluf M.P., Filho O.G., Filho H.M., Silvarolla M.B. (2000). Breeding and biotechnology of coffee. Coffee Biotechnology and Quality, Proceedings of the 3rd International Seminar on Biotechnology in the Coffee Agro-Industry, Londrina, Brazil.

[45] Geromel C., Ferreira L.P., Bottcher A., Pot D., Pereira L.F.P., Leroy T., Vieira L.G.E., Mazzafera P., Marraccini P. (2008). Sucrose metabolism during fruit development in *Coffea racemosa*. Ann. Appl. Biol..

[46] Costa D.C., Souza B.H., Carvalho C.H., Guerreiro Filho O. (2025). Characterization and levels of resistance in *Coffea arabica* × *Coffea racemosa* hybrids to *Leucoptera coffeella*. J. Pest. Sci..

[47] Liu C., Sack L., Li Y., Zhang J., Yu K., Zhang Q., He N., Yu G. (2023). Relationships of stomatal morphology to the environment across plant communities. Nat. Commun..

[48] Saridis P., Georgiadou X., Shtein I., Pouris J., Panteris E., Rhizopoulou S., Constantinidis T., Giannoutsou E., Adamakis I.-D.S. (2022). Stomata in Close Contact: The Case of *Pancratium maritimum* L. (Amaryllidaceae). Plants.

[49] Aguiar T.V., Sant’Anna-Santos B.F., Azevedo A.A., Ferreira R.S. (2007). Anati Quanti: Software de análises quantitativas para estudos em anatomia vegetal. Planta Daninha.

[50] Castro E.M., Pereira F.J., Paiva R. (2009). Histologia Vegetal: Estrutura e Função dos Órgãos Vegetativos.

[51] R Core Team (2021). R: A Language and Environment for Statistical Computing.

